# Cardiopulmonary Exercise Testing Identifies Treatable ATTR Cardiac Amyloidosis: Beyond Natriuretic Peptides

**DOI:** 10.1016/j.jaccas.2026.110006

**Published:** 2026-08-26

**Authors:** Sara Rioja-Abad, Ramon Andion-Ogando, Maria Perez-Diaz, Cristina Barinaga-Martin, Jordi Candela-Ferre, Maria Jesus Rollan-Gomez, Ignacio Hernandez-Gonzalez, Maria del Mar de la Torre-Carpente, Hector Garcia-Pardo, Juan Carlos Muñoz-San Jose

**Affiliations:** Department of Cardiology, Rio Hortega University Hospital, Valladolid, Spain

**Keywords:** cardiomyopathy, diastolic heart failure, imaging

## Abstract

**Background:**

Transthyretin cardiac amyloidosis (ATTR-CM) is a progressive infiltrative cardiomyopathy where early disease-modifying therapy improves outcomes. However, treatment access may be restricted by biomarker thresholds not reflecting true functional impairment.

**Case Summary:**

A 73-year-old man with exertional dyspnea (NYHA functional class II) and confirmed wild-type ATTR-CM did not meet reimbursement criteria for tafamidis because N-terminal pro–B-type natriuretic peptide was below the required threshold (≥600 pg/mL), despite impaired functional capacity on cardiopulmonary exercise testing (CPET). Acoramidis was obtained through expanded access. At 6-month follow-up, the patient showed clinical stability and improved exercise capacity.

**Discussion:**

Fixed N-terminal pro–B-type natriuretic peptide cutoffs may exclude patients with early-stage heart failure who would benefit from treatment. CPET helps identify therapy candidates below biomarker thresholds.

**Take-Home Messages:**

CPET detects functional impairment in ATTR-CM even when natriuretic peptides remain low. Early transthyretin-stabilizer initiation in NYHA functional class I-II yields better outcomes; treatment eligibility should integrate functional assessment beyond biomarkers alone.

## Visual Summary

**Figure:**
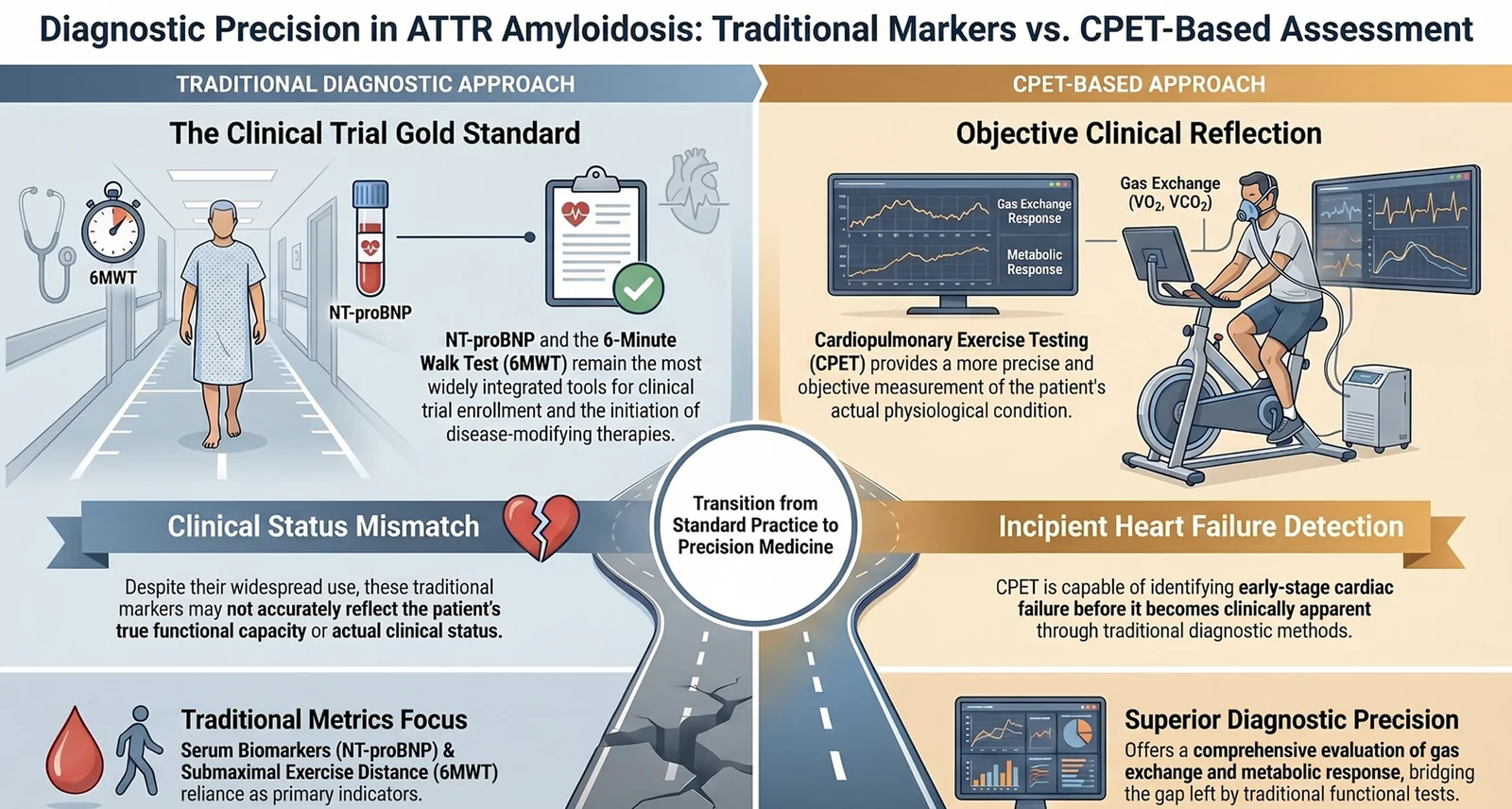

## History of Presentation: How the Patient Presented, Physical Examination

A 73-year-old man was referred to the cardiology clinic because of progressive exertional dyspnea and chest pain. Symptoms were compatible with NYHA functional class II.

He denied orthopnea, paroxysmal nocturnal dyspnea, syncope, or palpitations. On physical examination, blood pressure was 145/78 mm Hg, heart rate 75 beats/min, and oxygen saturation 95% on room air. Cardiac auscultation revealed regular rhythm with normal heart sounds and no murmurs. Pulmonary examination showed mild diffuse wheezing. The abdomen was soft without ascites. There was no peripheral edema in the lower extremities.

## Past Medical History

The patient had previous history of well-controlled arterial hypertension, obesity, former smoker, and dyslipidemia as cardiovascular risk factors. He was treated with enalapril 20 mg once daily, lercanidipine 10 mg once daily, and rosuvastatin 5 mg once daily, He underwent bilateral carpal tunnel surgery several years earlier. No other relevant medical history was reported.

## Differential Diagnosis

In a patient with multiple cardiovascular risk factors presenting with exertional dyspnea, ischemic heart disease and chronic obstructive pulmonary disease must be excluded as a potential cause of symptoms. The finding of left ventricular hypertrophy (≥12 mm) on cardiac imaging together with 2 additional red flags (heart failure with preserved ejection fraction in patients older than 65 years and bilateral carpal tunnel syndrome) should invite us to rule out cardiac amyloidosis.

## Investigations

Electrocardiogram showed sinus rhythm at 67 beats/min with pseudoinfarction pattern and low voltages. Chest radiography demonstrated mild pulmonary vascular redistribution without pleural effusion. Laboratory testing revealed impaired renal function (creatinine 1.7 mg/dL, estimated glomerular filtration rate 39 mL/min/1.73 m^2^), N-terminal pro–B-type natriuretic peptide (NT-proBNP) of 418 pg/mL, and high-sensitivity cardiac troponin I was 23 pg/mL (limit 19.8 pg/mL). Serum free light-chain assay revealed mildly increased free kappa light chains and abnormal kappa/lambda ratio, without evidence of monoclonal gammopathy on serum or urine immunofixation studies, arguing against AL amyloidosis. Exercise stress echocardiography was negative for inducible ischemia, and coronary computed tomography angiography demonstrated normal coronary arteries. Bone scintigraphy demonstrated grade 3 myocardial uptake. Genetic testing was negative for pathogenic transthyretin variants, confirming wild-type transthyretin amyloid cardiomyopathy (ATTR-CM). Cardiac magnetic resonance imaging showed a nondilated left ventricle with concentric hypertrophy (basal septum thickness 18 mm) and preserved systolic function (Figure 1). The right ventricle was hypertrophic but not dilated, with preserved contractility. Tissue characterization revealed diffuse late gadolinium enhancement with prolonged T1 values and increased extracellular volume, findings consistent with cardiac amyloidosis. Functional evaluation showed a 6-minute walk distance of 320 m.

**Figure 1:**
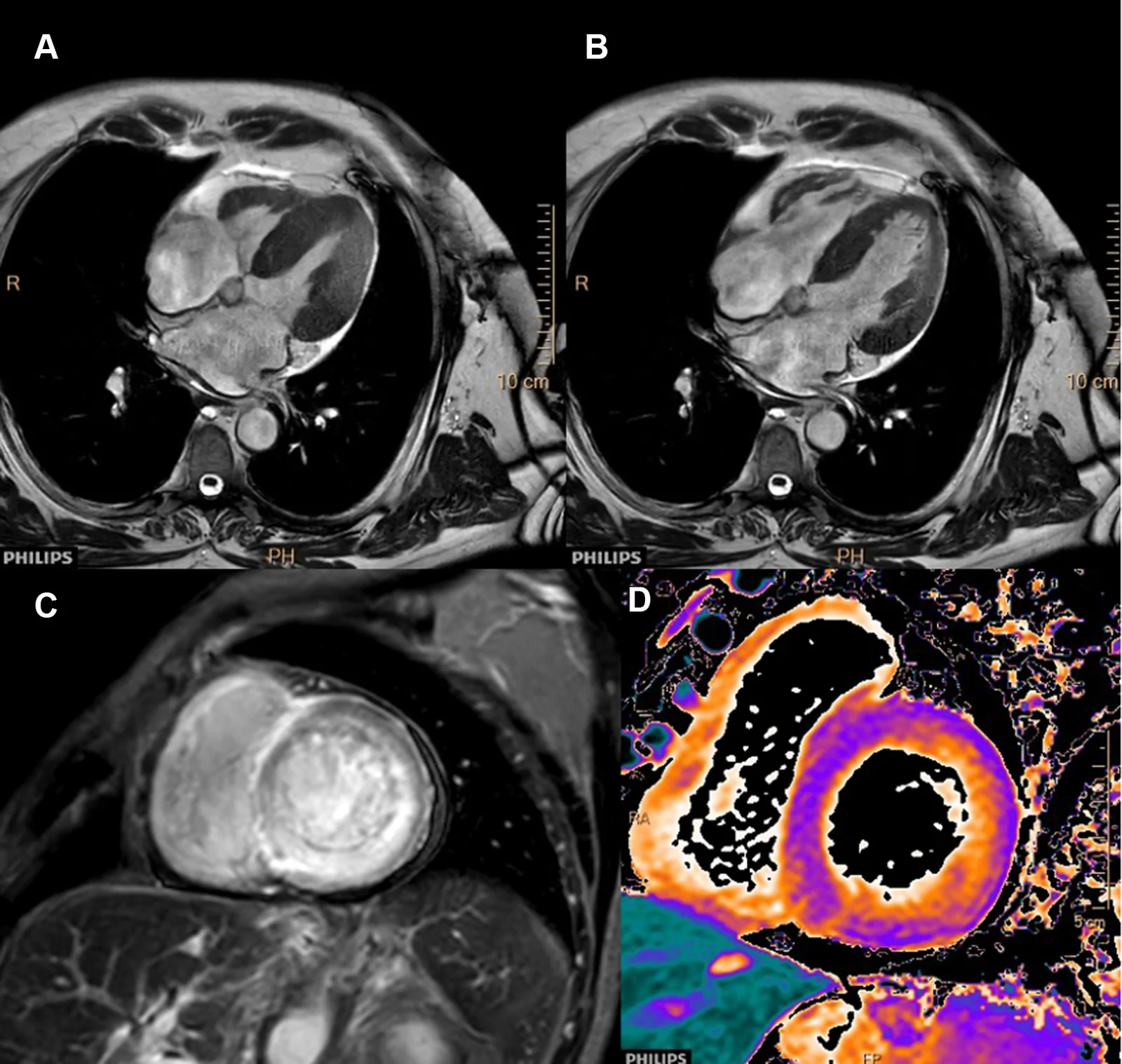
Cardiac Magnetic Resonance Imaging (A and B) Cine imaging 4-chamber view showing severe concentric hypertrophy. (C) Late gadolinium enhancement accompanied by a difficulty in nulling the myocardium. (D) Native T1 mapping demonstrating diffusely elevated myocardial T1 values.

Cardiopulmonary exercise testing (CPET) (Figure 2) demonstrated reduced peak oxygen consumption (16.7 mL/kg/min; 79% predicted) (Figure 3) and increased ventilatory inefficiency (ventilatory equivalent for carbon dioxide [VE/VCO_2_] slope 37) (Figure 3), corresponding to Weber class B and ventilatory class III, consistent with heart failure physiology.

**Figure 2:**
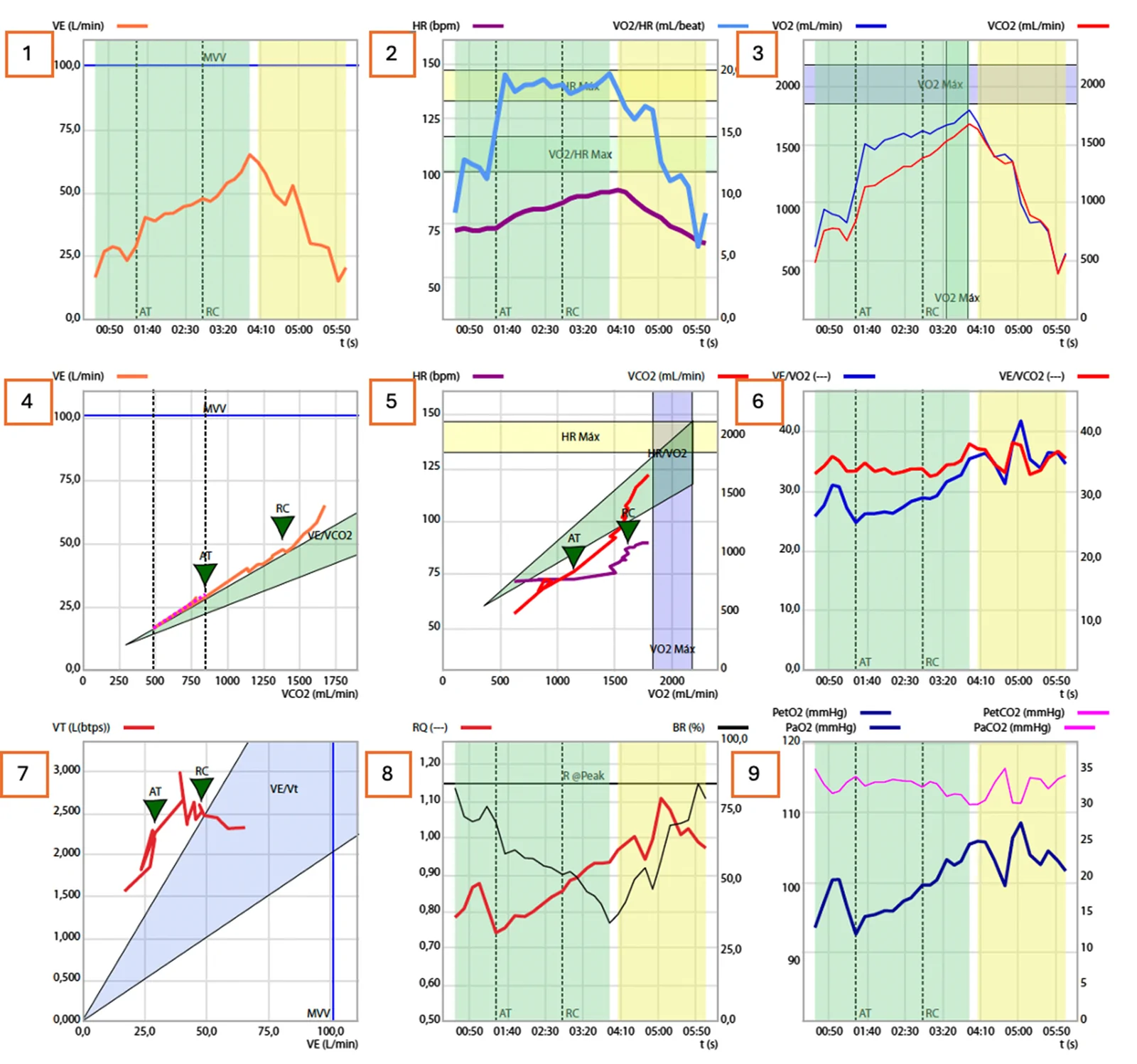
Cardiopulmonary Exercise Testing in a Patient With Heart Failure (1) (VE vs Watts): Shows a rapid increase in minute ventilation (orange diamonds) relative to the external work (green line), suggesting and early and exaggerated ventilatory drive. (2) Demonstrates a blunted oxygen pulse trajectory (blue circles), indicating an inability to augment stroke volume. The heart rate (purple curve) rises rapidly to compensate for the fixed stroke volume characteristic of infiltrative restrictive physiology. (3) Reveals a reduced peak oxygen consumption (VO_2_) (blue diamonds), classifying the patient as Weber class B. (4) Displays a markedly elevated ventilatory equivalent for carbon dioxide (VE/VCO_2_) slope, representing significant ventilatory inefficiency (Class III). (5) The V-slope analysis identifies an early anaerobic threshold, indicating that systemic oxygen delivery fails to meet metabolic demands at low exercise intensities. (6) Shows high nadir values for ventilatory equivalents, reinforcing the presence of subclinical pulmonary congestion. (7) A peak respiratory exchange ratio validates a maximal effort. (8) Shows a pathological decline in end-tidal partial pressure of carbon dioxide (PETCO2), indicating severe ventilation-perfusion mismatch and high physiological dead space. (9) Illustrates a rapid, shallow breathing pattern due to the limited ability to increase tidal volume (VT) in the setting of restrictive mechanics. HR = heart rate; PaO_2_ = partial pressure of oxygen; PaCO_2_ = partial pressure of carbon dioxide; PETCO_2_ = end-tidal partial pressure of carbon dioxide; RQ = respiratory quotient.

**Figure 3:**
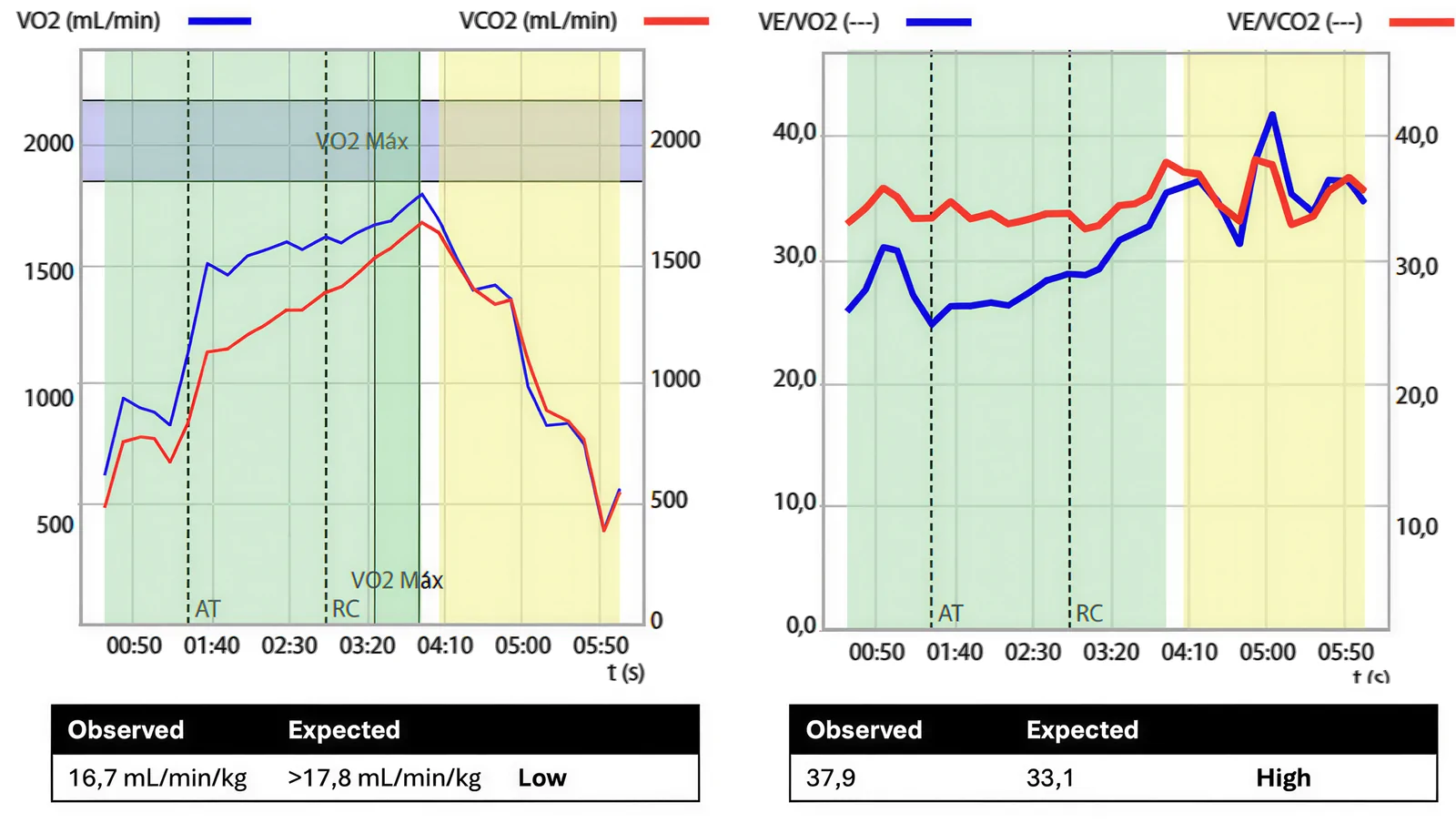
CPET in Patient With Heart Failure Left: Cardiopulmonary exercise testing (CPET) demonstrating reduced peak oxygen consumption (VO_2_) and early anaerobic threshold (AT), reflecting impaired cardiac reserve in cardiac amyloidosis. Right: Restrictive physiology with reduced exercise capacity and ventilatory inefficiency (elevated ventilatory equivalent for carbon dioxide [VE/VCO_2_] slope).

## Management (Medical/Interventions)

The patient was diagnosed with wild-type ATTR-CM with heart failure with preserved ejection fraction. Despite symptomatic heart failure and confirmed ATTR-CM, the patient did not meet the reimbursement criteria for tafamidis because natriuretic peptide levels were below the required threshold. Given the clear functional impairment documented by CPET, treatment with the transthyretin stabilizer acoramidis was requested through an expanded access program and subsequently initiated.

## Outcomes and Follow-Up

After 6 months of treatment with acoramidis, the patient demonstrated a favorable clinical course, stable in NYHA functional class II, with no hospitalizations, and decreasing NT-proBNP levels. Quality-of-life questionnaire scores and 6-minute walk test improved. Treatment adherence was optimal, and no drug-related adverse effects were observed.

## Discussion

Transthyretin cardiac amyloidosis (ATTR-CM) is a progressive infiltrative cardiomyopathy characterized by extracellular deposition of misfolded protein in which early diagnosis and timely initiation of disease-modifying therapies are critical determinants of prognosis.^1^, ^2^, ^3^ It is important to note at the outset that the reimbursement restrictions described in this report are specific to the Spanish regulatory framework and do not reflect prescribing practice in the United States. U.S. clinicians are therefore not subject to the biomarker-driven access restrictions encountered in this case, and the clinical dilemma described herein should be interpreted within its specific European regulatory context. In Spain, the reimbursement criteria approved by the Spanish Ministry of Health require NT-proBNP levels above 600 pg/mL. The present case illustrates a clinically relevant dilemma: a symptomatic patient with confirmed ATTR-CM and objective functional impairment who did not meet reimbursement criteria for tafamidis because natriuretic peptide levels were below a threshold, which may occur despite established disease in the presence of many noncardiac comorbidities. Although this cutoff is derived from the ATTR-ACT (Tafamidis in Transthyretin Cardiomyopathy Clinical Trial),^4^ it may not adequately reflect the patients’ clinical status. This scenario highlights the limitations of relying on absolute biomarker cutoffs^5^ and underscores the need for a more comprehensive functional assessment,^6^ particularly through CPET.^7^, ^8^, ^9^, ^10^

## Conclusions

CPET is a valuable tool for detecting and quantifying functional impairment in transthyretin cardiac amyloidosis and provides important prognostic and therapeutic information. CPET can reveal clinically meaningful cardiopulmonary limitations even when resting biomarkers appear relatively preserved.

In the European setting, rigid natriuretic peptide thresholds to determine eligibility for transthyretin-stabilizing therapies may therefore not lead to patients with early-stage disease. A more integrated approach combining biomarkers, imaging, and objective functional assessment may allow for earlier identification of patients who may benefit from disease-modifying therapy.

**Take-Home Messages**

• CPET provides an integrated assessment of functional capacity and may reveal clinically significant impairment in patients with ATTR-CM even when natriuretic peptide levels are relatively low.
• Disease-modifying therapies have the greatest impact when initiated early; in this way, treatment decisions should therefore not rely solely on biomarker thresholds when other objective evidence of functional limitation is present.

**Visual Summary:**
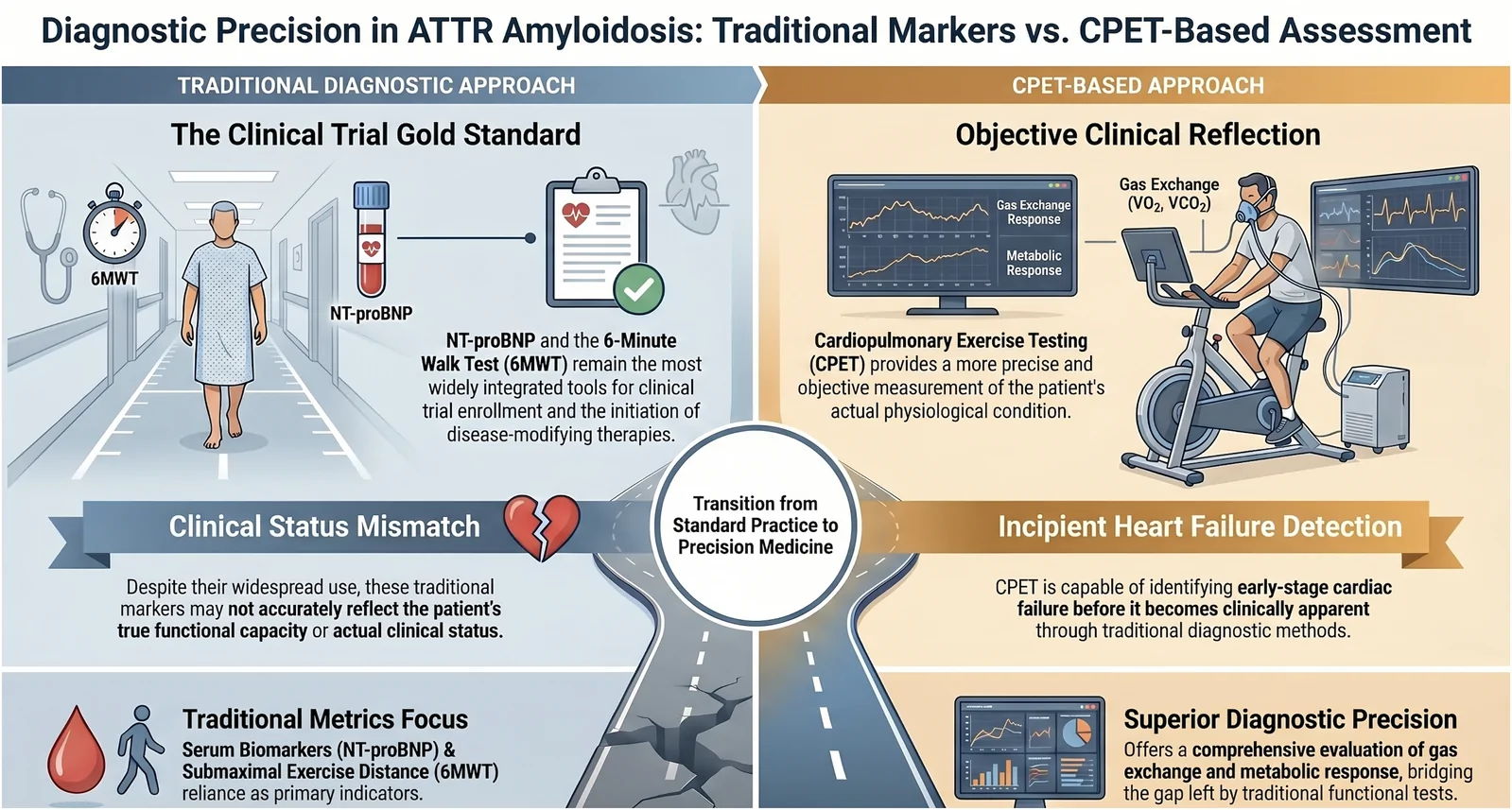
Comparison Between the Traditional Approach and Cardiopulmonary Exercise Testing–Based Assessment Although traditional markers (6-minute walk test [6MWT], N-terminal pro–B-type natriuretic peptide [NT-proBNP]) are trial standards, they can lead to a clinical status mismatch by failing to reflect true functional capacity. In contrast, cardiopulmonary exercise testing (CPET) provides an objective clinical reflection of physiological status, enabling the detection of incipient heart failure. ATTR amyloidosis = transthyretin amyloidosis; VCO_2_ = volume of carbon dioxide; VO_2_ = oxygen consumption.

## Funding Support and Author Disclosures

The authors have reported that they have no relationships relevant to the contents of this paper to disclose.
